# The Story of the Insane from Year to Year

**Published:** 1890-11-15

**Authors:** 


### The Story of the Insane from Year to Year.

### THE LANCASTER COUNTY ASYLUM AT LANCASTER MOOR.

We have often said that most of our borough asylums labour under the disadvantage of being too small, and many of our county asylums have just the opposite fault. Among these is the asylum at Lancaster, which closed the year 1889 with 1,903 patients. There were 303 admissions, 263 discharges, and 132 deaths. This is a very respectable "turnover," and we find that it yields a percentage of 45·54 recoveries and 6·84 deaths. Dr. Cassidy gives us an interesting piece of biography. A man named O'Neil (with fifteen aliases) was readmitted on July 6th, and recognised as a maligner and swindler. After being sent from Lancaster Asylum on former occasions, he had gone to relatives of patients and obtained money from them. On one occasion he had written a letter in Dr. Cassidy's name to a tailor in Lancaster to supply him with a suit of clothes. He obtained the clothes and disappeared. O'Neil was found stupid with drink by the police at Garstang, and sent to the asylum. Dr. Cassidy sent him back to gaol. He was subsequently tried at Preston, and sentenced to five years' penal servitude. Curious waifs and strays are often to be found in our county asylums, but we hope they are not often so bad as O'Neil. Thirty of the deaths were due to phthisis, and Dr. Cassidy thinks this number is too high in a death total of 132. He accepts the view that phthisis is caused by the tubercle bacillus, and he acts upon the view by separating the consumptives from the rest of the sick, keeping the atmosphere of the rooms charged with the vapour of eucalyptus and peppermint. Without wishing to make invidious distinctions, we may record our opinion that Dr. Cassidy's report is one of the ablest of the year 1889.

